# Safety and efficacy of abiraterone acetate plus prednisolone in patients with castration-resistant prostate cancer: a prospective, observational, post-marketing surveillance study

**DOI:** 10.1093/jjco/hyab077

**Published:** 2021-05-29

**Authors:** Yosuke Koroki, Keiichiro Imanaka, Yukiko Yasuda, Sayuri Harada, Akiko Fujino

**Affiliations:** Medical Affairs, Janssen Pharmaceutical K.K., Tokyo, Japan; Clinical Science, Janssen Pharmaceutical K.K., Tokyo, Japan; Japan Safety and Surveillance, Janssen Pharmaceutical K.K., Tokyo, Japan; Japan Safety and Surveillance, Janssen Pharmaceutical K.K., Tokyo, Japan; Japan Safety and Surveillance, Janssen Pharmaceutical K.K., Tokyo, Japan

**Keywords:** castration-resistant prostate cancer, abiraterone acetate, prednisolone, anti-androgen, CYP17 inhibition, post-marketing surveillance

## Abstract

**Background:**

Abiraterone acetate plus prednisolone is approved to treat patients with castration-resistant prostate cancer. This study evaluated the safety and efficacy of abiraterone acetate plus prednisolone in castration-resistant prostate cancer patients with or without previous chemotherapy in a real-world setting in Japan.

**Methods:**

This study was an observational, prospective, post-marketing surveillance. Castration-resistant prostate cancer patients, who initiated abiraterone acetate after its approval in Japan, were enrolled. Data were collected during an observation period of 12 months and a follow-up period of another 12 months. Adverse events and adverse drug reactions were evaluated for safety. Prostate-specific antigen levels and overall survival were evaluated for efficacy.

**Results:**

From 141 participating institutions, 497 patients were registered: 492 patients including 180 chemotherapy-naïve, 311 chemotherapy-experienced and one off-label-use patient received abiraterone and were evaluated for safety. Adverse events were observed in 225/492 patients (45.7%), adverse drug reactions in 131/492 patients (26.6%) and serious adverse drug reactions in 61/492 patients (12.4%). The most commonly observed adverse drug reaction was abnormal hepatic function (6.5%), followed by hypokalemia (3.0%) and decreased appetite (2.0%). At week 12, 110/432 patients (25.5%) achieved ≥50% decrease from baseline in prostate-specific antigen, and the proportion was higher in chemotherapy-naïve patients (56/161 patients; 34.8%) compared with chemotherapy-experienced patients (54/271 patients; 19.9%, *P* < 0.001). Survival rates at 24 months were 68.3% (295/432 patients), 73.9% (119/161 chemotherapy-naïve patients) and 64.9% (176/271 chemotherapy-experienced patients).

**Conclusions:**

This large-scale, real-world, post-marketing surveillance study confirmed the safety and efficacy of abiraterone acetate plus prednisolone in Japanese castration-resistant prostate cancer patients with or without previous chemotherapy.

## Teaser

This large-scale, real-world, post-marketing surveillance study in Japan confirmed the safety and efficacy of abiraterone acetate plus prednisolone in castration-resistant prostate cancer patients with or without previous chemotherapy.

## Introduction

Prostate cancer is common. Among males, it is the most commonly diagnosed cancer in 105 countries worldwide including most American and European countries (1). In most patients, initial androgen deprivation therapy effectively regresses the tumor, but castration resistance inevitably emerges and the tumor becomes resistant to androgen deprivation therapy. Various mechanisms of how castration-resistant prostate cancer (CRPC) develops have been reported: e.g. androgen receptor amplification, increased androgen receptor sensitivity and increased androgen synthesis (2). Cytotoxic chemotherapy using docetaxel has been widely used to treat patients with CRPC and has demonstrated benefit in extending the median overall survival (OS) in patients with CRPC (3,4). In addition, a new taxane agent, cabazitaxel, was recently developed and used for cytotoxic chemotherapy (5).

Abiraterone acetate is a prodrug of abiraterone that inhibits androgen synthesis in the testis, adrenal glands and tumor tissues by selectively inhibiting the CYP17 enzyme and offers a new approach to treat metastatic CRPC (mCRPC) patients. It has been approved and used since 2014 in Japan.

Several clinical studies have demonstrated the favourable efficacy and tolerable safety profile of abiraterone acetate plus prednisolone treatment. In the COU-AA-302 phase III trial with 1088 mCRPC patients without previous chemotherapy, abiraterone acetate plus prednisone or prednisolone significantly extended the median OS compared with placebo (34.7 vs. 30.3 months; hazard ratio, 0.81; *P* = 0.0033) (6). Another phase III trial, COU-AA-301, reported that abiraterone extended the median OS compared with placebo in mCRPC patients previously treated with docetaxel (14.8 vs. 10.9 months; hazard ratio, 0.65; *P* < 0.001) (7). Abiraterone acetate plus prednisone was first approved by the Food and Drug Administration in 2011 and is approved to date in >100 countries including Japan. Combination therapy of abiraterone acetate plus prednisone or prednisolone is recommended as the first-line treatment for mCRPC patients by the European (8) and Japanese guidelines (9).

Known adverse events (AEs), including fluid retention, hypokalemia and hypertension, are associated with elevated levels of mineral corticoid due to CYP blockade and are mitigated by concurrent prednisone/prednisolone treatment (7,10). Furthermore, abiraterone is associated with cardiac disorders and liver dysfunction (7,10), which warrants the long-term monitoring of this drug in a real-world setting.

The objective of this post-marketing surveillance study was to assess the safety and efficacy of abiraterone acetate plus prednisolone in patients with CRPC in a real-world setting in Japan.

## Patients and methods

### Ethics

This study was conducted in accordance with the Japanese Ministerial Ordinance on Good Post-marketing Study Practice for Drugs (MHLW Ordinance No. 171 of 2004). In this study, written informed consent from patients was not necessary because the treatment and investigations were within general practice and all data were anonymized.

### Study design

This was an observational, prospective, post-marketing surveillance study. Patients were enrolled from 16 September 2014 to 31 August 2016, and data were collected from 16 September 2014 to 28 February 2019.

Patients with CRPC, whose abiraterone acetate plus prednisolone treatment was planned for the first time, were eligible. Abiraterone acetate was to be administered orally in the fasting state once daily (ZYTIGA®, Janssen Pharmaceutical K.K., Tokyo, Japan) in combination with prednisolone. The observation period was defined as 12 months (52 weeks) from the starting day of the treatment. The follow-up period was defined as the period starting from the day after the last day of the observation period and ending up to 12 months (52 weeks) later.

### Data collection

Data were recorded in a case-report form by study physicians using an internet-based electronic data capturing system from the initiation of abiraterone acetate plus prednisolone treatment until the end of the observation period. Recorded information included background characteristics including previous use of chemotherapy, Gleason score, presence and sites of metastasis, previous treatment for prostate cancer; abiraterone treatment during the observation period including dose, treatment period and reasons of discontinuation; concomitant medications; abiraterone treatment at the end of the observation period; clinical outcomes and reasons for discontinuation, if applicable; serum levels of prostate-specific antigen (PSA); AEs and AE-related medications and clinical outcomes during the follow-up period. All data were anonymized and collected under a contract between participating institutions and Janssen Pharmaceutical K.K.

### Safety evaluation

Patients who violated the contract or the study protocol were excluded from the safety analyses. All AEs, including disease progression, were collected from all patients who received abiraterone. The AE terms, date of onset, seriousness, intervention, date of outcome, date outcome confirmed, causal relationship, possible causative treatment or factors were recorded. AEs for which a causal relationship to abiraterone could not be excluded were defined as adverse drug reactions (ADRs).

From the safety profiles observed in pre-approval clinical studies, ADRs associated with elevated mineral corticoid levels by CYP receptor blockade such as hypertension, fluid retention and edema and hypokalemia as well as cardiac disorders, hepatotoxicity-related events and osteoporosis and osteoporotic fractures were often observed and therefore important to monitor. Thus, as in previous studies, the following ADRs in this study were defined as ADRs of special interest: hypertension, fluid retention and edema (including edema and edema peripheral), hypokalemia, cardiac disorders (including loss of consciousness, acute myocardial infarction, angina pectoris, chest pain), hepatotoxicity-related ADRs (including hepatic function abnormal, hepatotoxicity, liver disorder, drug-induced liver injury, alanine aminotransferase increased, aspartate aminotransferase increased, liver function test abnormal, hepatic enzyme increased) and osteoporosis and osteoporotic fractures. These ADRs of special interest as well as the use of abiraterone in patients with hepatic impairment were analysed as priority survey items.

### Efficacy evaluation

Serum PSA was used to assess drug efficacy. Patients who did not have PSA levels at baseline and at 12 weeks or at least once during the observation period were excluded from the efficacy analysis set. Patients with a ≥50% reduction of PSA from baseline at 12 weeks after treatment initiation were considered responders. The proportion of responders in the efficacy analysis set was defined as the response rate. The response rate and OS were calculated for all patients in the efficacy analysis and for patients in two subgroups based on previous chemotherapy treatments: patients who had no previous treatment with chemotherapy (chemotherapy-naïve) and those who had previously received chemotherapy (chemotherapy-experienced). The OS rate was defined as the proportion of patients who were alive at the end of the follow-up period or the time of censoring.

### Statistics

Patient disposition, safety data and efficacy data including PSA levels were summarized descriptively. Response rates were compared between the chemotherapy-naïve and chemotherapy-experienced groups using Fisher’s exact test. Patients were categorized according to the period of anti-androgen therapy and response rates were compared using the χ^2^ test. The Kaplan–Meier method was applied for OS.

We planned to enrol 420 patients, including 170 chemotherapy-naïve and 250 chemotherapy-experienced patients. In phase II clinical studies conducted in Japan (11,12), the least frequent AEs of special interest were found in 1/95 patients (1.05%, ventricular tachycardia, edema and bradycardia). Therefore, 300 patients would be necessary to detect at least one AE with a ≥95% probability. In the global phase III studies, the OS rate for 12 months in patients without previous chemotherapy was 91.2%, and it was 61.1% in patients with previous chemotherapy (unpublished data). Based on these studies, we estimated that 90% of chemotherapy-naïve patients and 60% of chemotherapy-experienced patients would remain under observation at the12-month time point after the initiation of treatment. To obtain 300 patients after the 12-month observation period, including 150 patients for each chemotherapy cohort, a total of 420 patients including 170 chemotherapy-naïve and 250 chemotherapy-experienced patients were considered necessary.

## Results

### Patient demographic data and use of abiraterone

**Figure 1.**
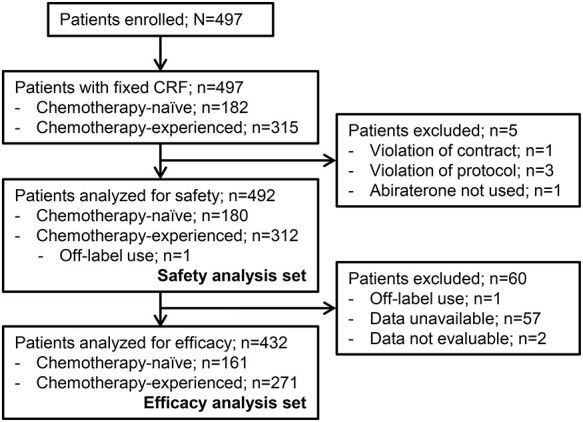
Patient flow. CRF, case-report form collected by electronic data-capturing system.

During the study period, 497 patients with CRPC were recruited in 141 participating institutions and registered. Of those, safety was analysed in 492 patients, including 180 chemotherapy-naïve and 312 chemotherapy-experienced patients, and one off-label use was included in chemotherapy-experienced patients. Efficacy was analysed in 432 patients, including 161 chemotherapy-naïve and 271 chemotherapy-experienced patients. The patient flow is shown in Figure 1.

Patient demographics are summarized in Table 1. The median age was 76.0 (range, 52–97) years and most patients were ≥65 years of age (92.9%). Metastasis was present in 395/492 patients (80.3%). The most frequent metastatic site was bone (329/492 patients, 66.9%) followed by lymph nodes (198/492, 40.2%). Most patients (482/492 patients, 98.0%) had previously used anti-androgens, including bicalutamide (464/492, 94.3%), flutamide (342/492, 69.5%) and enzalutamide (96/492, 19.5%). Median PSA values at baseline were 19.0 (range, 0–3738) ng/ml in the overall population, 16.0 (0–1819) ng/ml in chemotherapy-naïve patients and 23.7 (0–3738) ng/ml in chemotherapy-experienced patients (Table 1). The patient demographic data were similar between chemotherapy-naïve and chemotherapy-experienced cohorts.

**Table 1:** Patient demographics (safety analysis set, *N* = 492)

Demographics	Total, *N* = 492, *n* (%)	Chemotherapy-naïve, *N* = 180, *n* (%)	Chemotherapy-experienced, *N* = 311, *n* (%)
Sex, male	492 (100.0)	180 (100.0)	311 (100.0)
Age (year)			
Mean ± SD	75.5 ± 7.4	76.8 ± 7.2	74.7 ± 7.4
Median (range)	76.0 (52–97)	78.0 (56–90)	75.0 (52–97)
<65	35 (7.1)	9 (5.0)	25 (8.0)
≥65	457 (92.9)	171 (95.0)	286 (92.0)
Gleason score			
2–7	84 (17.1)	30 (16.7)	54 (17.4)
8–10	348 (70.7)	124 (68.9)	223 (71.7)
Unknown	60 (12.2)	26 (14.4)	34 (10.9)
Previous surgery^a^			
No	402 (81.7)	149 (82.8)	252 (81.0)
Yes	90 (18.3)	31 (17.2)	59 (19.0)
Radical prostatectomy	42 (8.5)	11 (6.1)	31 (10.0)
Transurethral resection of the prostate	12 (2.4)	4 (2.2)	8 (2.6)
Other	43 (8.7)	17 (9.4)	26 (8.4)
Previous radiotherapy^a^			
No	368 (74.8)	147 (81.7)	220 (70.7)
Yes	124 (25.2)	33 (18.3)	91 (29.3)
Prostate	76 (15.4)	21 (11.7)	55 (17.7)
Bone	60 (12.2)	15 (8.3)	45 (14.5)
Other	6 (1.2)	1 (0.6)	5 (1.6)
Previous anti-androgens^a^			
No	10 (2.0)	1 (0.6)	8 (2.6)
Yes	482 (98.0)	179 (99.4)	303 (97.4)
Bicalutamide	464 (94.3)	174 (96.7)	290 (93.2)
Flutamide	342 (69.5)	113 (62.8)	229 (73.6)
Enzalutamide	96 (19.5)	32 (17.8)	64 (20.6)
Chlormadinone acetate	54 (11.0)	18 (10.0)	36 (11.6)
Ethinylestradiol	41 (8.3)	12 (6.7)	29 (9.3)
Fosfestrol	1 (0.2)	0 (0.0)	1 (0.3)
Previous chemotherapy^a^			
No	180 (36.6)	180 (100.0)	0 (0.0)
Yes	312 (63.4)	0 (0.0)	311 (100.0)
Estramustine phosphate sodium hydrate	207 (42.1)	N/A	207 (66.6)
Docetaxel hydrate	177 (36.0)	N/A	176 (56.6)
Other	26 (5.3)	N/A	26 (8.4)
Metastasis^a^			
Absent	97 (19.7)	37 (20.6)	60 (19.3)
Present	395 (80.3)	143 (79.4)	251 (80.7)
Soft tissue	4 (0.8)	1 (0.6)	3 (1.0)
Lymph node	198 (40.2)	78 (43.3)	120 (38.6)
Bone	329 (66.9)	113 (62.8)	215 (69.1)
Liver	11 (2.2)	4 (2.2)	7 (2.3)
Lung	28 (5.7)	11 (6.1)	17 (5.5)
Other	4 (0.8)	1 (0.6)	3 (1.0)
Concomitant disease^a^			
No	296 (60.2)	99 (55.0)	196 (63.0)
Yes	196 (39.8)	81 (45.0)	115 (37.0)
Kidney disorder	14 (2.8)	6 (3.3)	8 (2.6)
Liver disorder	11 (2.2)	2 (1.1)	9 (2.9)
Baseline PSA level (ng/ml)			
Median (range)	19.0 (0–3738)	16.0 (0–1819)	23.7 (0–3738)
Unknown	8	1	7
Abiraterone treatment duration (week)			
Median (range)	20.9 (0.9–81.4)	27.6 (0.9–57.3)	18.5 (1.7–81.4)
Abiraterone daily dose at treatment initiation (mg)			
Mean ± SD	968.5 ± 116.3	968.1 ± 121.2	968.7 ± 113.7
1000	455 (92.5)	167 (92.8)	287 (92.3)
750	13 (2.6)	4 (2.2)	9 (2.9)
500	23 (4.7)	8 (4.4)	15 (4.8)
250	1 (0.2)	1 (0.6)	0 (0.0)
Abiraterone daily dose (mg)			
Mean ± SD	964.4 ± 112.3	964.8 ± 112.9	964.1 ± 112.3
1,000	433 (88.0)	159 (88.3)	273 (87.8)
750–<1,000	29 (5.9)	9 (5.0)	20 (6.4)
500–<750	30 (6.1)	12 (6.7)	18 (5.8)
250–<500	0 (0.0)	0 (0.0)	0 (0.0)
<250	0 (0.0)	0 (0.0)	0 (0.0)

^a^Multiple answers possible.

PSA, prostate-specific antigen; SD, standard deviation; N/A, not applicable.

By the end of the observation period, 377/492 patients (76.6%) had discontinued the treatment. The most frequent reason for discontinuation was unsatisfactory efficacy (253/377 patients, 67.1%) followed by AEs (80/377, 21.2%, Table 2). The median duration of abiraterone treatment was 20.9 (range, 0.9–81.4) weeks. The duration of abiraterone treatment is summarized in Table S1.

**Table 2:** Reason for discontinuation at the end of the observation period (safety analysis set, *N* = 492)

Reason for discontinuation	Number of patients discontinued, *n* (%)
Discontinued Efficacy unsatisfactory Adverse events Patient’s will Hospital change No hospital visit Other	377 (100.0) 253 (67.1) 80 (21.2) 20 (5.3) 11 (2.9) 3 (0.8) 10 (2.7)

### Safety

In the safety analysis set of 492 patients, 318 AEs were found in 225 patients (45.7%). Of these 225 patients, 160 experienced 205 serious AEs (160/492 patients, 32.5%), including 64 events in 49/180 chemotherapy-naïve patients (27.2%) and 141 events in 111/311 chemotherapy-experienced patients (35.7%). No AEs were observed in patient with off-label use (data not shown). The most frequently observed AE was progression of prostate cancer (75/492 patients, 15.2%), followed by abnormal hepatic function (32/492, 6.5%). AEs led to treatment discontinuation in 88/492 patients (17.9%) and dose reduction in 10/492 patients (2.0%). Frequent AEs leading to treatment modification are summarized in Table S2.

ADRs and serious ADRs are summarized in Table 3. Overall, 170 ADRs were observed in 131/492 patients (26.6%), including 63 events in 48/180 chemotherapy-naïve patients (26.7%) and 107 events in 83/311 chemotherapy-experienced (26.7%) patients. Serious ADRs comprised 77 events in 61/492 patients (12.4%), including 28 events in 21/180 chemotherapy-naïve patients (11.7%) and 49 events in 40/311 (12.9%) chemotherapy-experienced patients. The most frequently observed ADR was abnormal hepatic function (32/492 patients, 6.5%), followed by hypokalemia (15/492 patients, 3.0%).

**Table 3:** Summary of adverse drug reactions and those observed in ≥1% of all patients and those of special interest observed in at least one patient (safety analysis set, *N* = 492)

Adverse drug reactions	Serious adverse drug reaction, number of patients (%)			Adverse drug reaction of any grade, number of patients (%)		
	Total, *N* = 492	Chemotherapy-naïve, *N* = 180	Chemotherapy-experienced, *N* = 311	Total, *N* = 492	Chemotherapy-naïve, *N* = 180	Chemotherapy-experienced, *N* = 311
Any adverse drug reactions						
Number of patients (%)	61 (12.4)	21 (11.7)	40 (12.9)	131 (26.6)	48 (26.7)	83 (26.7)
Number of events (%)	77 (15.7)	28 (15.6)	49 (15.8)	170 (34.6)	63 (35.0)	107 (34.4)
Adverse drug reaction of special interest^a^						
Cardiac disorders	3 (0.6)	2 (1.1)	1 (0.3)	4 (0.8)	2 (1.1)	2 (0.6)
Hepatotoxicity-related ADRs	23 (4.7)	7 (3.9)	16 (5.1)	47 (9.6)	18 (10.0)	29 (9.3)
Neoplasms benign, malignant, or unspecified (including cysts and polyps)						
Prostate cancer	6 (1.2)	3 (1.7)	3 (1.0)	6 (1.2)	3 (1.7)	3 (1.0)
Metabolism and nutrition disorders						
Hypokalemia^b^	5 (1.0)	2 (1.1)	3 (1.0)	15 (3.0)	5 (2.8)	10 (3.2)
Decreased appetite	2 (0.4)	1 (0.6)	1 (0.3)	10 (2.0)	3 (1.7)	7 (2.3)
Nervous system disorders						
Loss of consciousness^c^	1 (0.2)	0 (0.0)	1 (0.3)	1 (0.2)	0 (0.0)	1 (0.3)
Cardiac disorders						
Acute myocardial infarction^c^	1 (0.2)	1 (0.6)	0 (0.0)	1 (0.2)	1 (0.6)	0 (0.0)
Angina pectoris^c^	1 (0.2)	1 (0.6)	0 (0.0)	1 (0.2)	1 (0.6)	0 (0.0)
Vascular disorders						
Hypertension^c^	2 (0.4)	0 (0.0)	2 (0.6)	7 (1.4)	4 (2.2)	3 (1.0)
Gastrointestinal disorders						
Nausea	0 (0.0)	0 (0.0)	0 (0.0)	5 (1.0)	0 (0.0)	5 (1.6)
Hepatobiliary disorders						
Hepatic function abnormal^e^	16 (3.3)	3 (1.7)	13 (4.2)	32 (6.5)	11 (6.1)	21 (6.8)
Hepatotoxicity^e^	3 (0.6)	1 (0.6)	2 (0.6)	5 (1.0)	2 (1.1)	3 (1.0)
Liver disorder^e^	0 (0.0)	0 (0.0)	0 (0.0)	2 (0.4)	1 (0.6)	1 (0.3)
Drug-induced liver injury^e^	1 (0.2)	0 (0.0)	1 (0.3)	1 (0.2)	0 (0.0)	1 (0.3)
General disorders and administration site conditions						
Chest pain^c^	0 (0.0)	0 (0.0)	0 (0.0)	1 (0.2)	0 (0.0)	1 (0.3)
Malaise	0 (0.0)	0 (0.0)	0 (0.0)	6 (1.2)	2 (1.1)	4 (1.3)
Edema peripheral^f^	0 (0.0)	0 (0.0)	0 (0.0)	2 (0.4)	2 (1.1)	0 (0.0)
Investigations						
Alanine aminotransferase increased^e^	2 (0.4)	2 (1.1)	0 (0.0)	4 (0.8)	2 (1.1)	2 (0.6)
Aspartate aminotransferase increased^e^	2 (0.4)	2 (1.1)	0 (0.0)	3 (0.6)	2 (1.1)	1 (0.3)
Liver function test abnormal^e^	1 (0.2)	1 (0.6)	0 (0.0)	2 (0.4)	2 (1.1)	0 (0.0)
Hepatic enzyme increased^e^	0 (0.0)	0 (0.0)	0 (0.0)	1 (0.2)	0 (0.0)	1 (0.3)

Events were coded using system organ classes (SOC) and preferred terms (PT) defined in MedDRA/J Ver. 22.0.

^a^Composite of multiple adverse drug reactions

^b^Adverse drug reaction of special interest: hypokalemia

^c^Adverse drug reaction of special interest: cardiac disorders

^d^Adverse drug reaction of special interest: hypertension

^e^Adverse drug reaction of special interest: hepatotoxicity-related ADRs

^f^Adverse drug reaction of special interest: fluid retention and edema

#### Priority survey items: ADRs of special interest

Regarding ADRs of special interest, seven events of hypertension were reported in 7/492 patients (1.4%), two cases of which were serious. For fluid retention and edema (edema peripheral), two events were reported in 2/492 patients (0.4%) and these were not serious. Osteoporosis and osteoporotic fractures were not observed during the study period.

**Figure 2.**
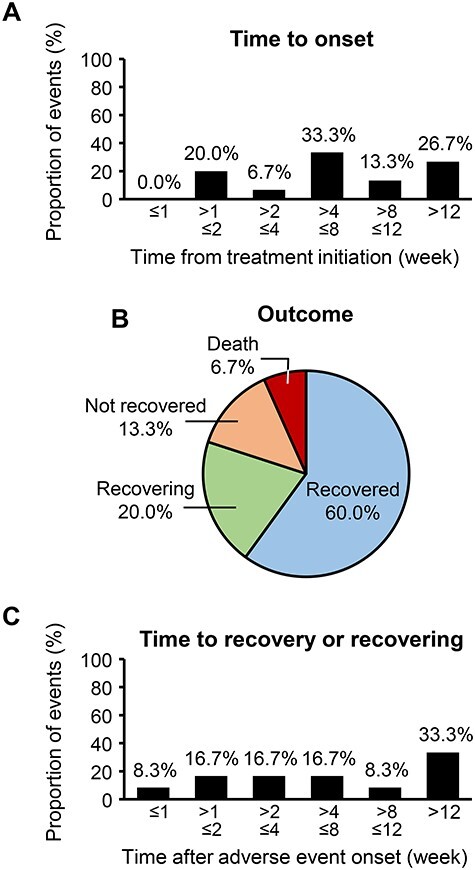
Adverse event of special interest: hypokalemia. (A) Time to onset of hypokalemia after treatment initiation in patients who showed hypokalemia (*N* = 15). (B) Outcome of hypokalemia (15 events in 15 patients). (C) Time from hypokalemia onset to recovery or recovering (12 events in 12 patients).

For hypokalemia, 15 events were observed in 15/492 patients (3.0%). Time to onset after treatment start is summarized in Figure 2A. Most events were recovered (9/15, 60.0%) or recovering (3/15, 20.0%; Fig. 2B). Regarding the recovered and recovering hypokalemia (12 events), time to recovery or recovering varied among the patients, but 4/12 patients (33.3%) recovered >12 weeks after onset (Fig. 2C). Hypokalemia led to abiraterone discontinuation in 4/15 patients (26.7%) and a dose reduction in 1/15 patients (6.7%); 10/15 patients (66.7%) continued without dose adjustment.

Cardiac disorders pre-specified as ADRs of special interest including loss of consciousness, acute myocardial infarction, angina pectoris and chest pain were observed in each one patient (1/492 patients, 0.2% each). The patient experienced acute myocardial infarction 69 days after the discontinuation of abiraterone and died same day. This 79-year-old patient had no medical history or concomitant disease. Another cause of death was progression of prostate cancer. The outcomes of remaining patients were either recovering or recovered.

**Figure 3.**
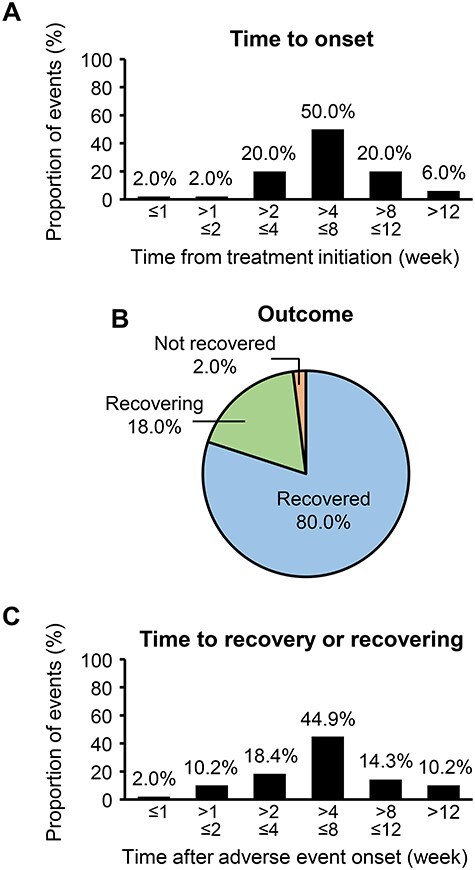
Adverse events of special interest: hepatotoxicity-related ADRs. (A) Time to onset of hepatotoxicity-related ADRs after treatment initiation (*N* = 47). (B) Outcome of hepatotoxicity-related ADRs (50 events in 47 patients). (C) Time from onset to recovery or recovering (49 events in 46 patients). ADR, adverse drug reaction.

For hepatotoxicity-related ADRs (including hepatic function abnormal, hepatotoxicity, liver disorder, drug-induced liver injury, alanine aminotransferase increased, aspartate aminotransferase increased, liver function test abnormal, hepatic enzyme increased), 50 events were reported for 47 patients (Table 3). Of these, 25 hepatotoxicity-related ADRs in 24/47 patients (51.1%) occurred at 4–8 weeks after treatment initiation (Fig. 3A). Of all hepatotoxicity-related ADRs, 49 events (98.0%) were recovered or were recovering (Fig. 3B). Time to recovery or recovering is summarized in Figure 3C. Abiraterone was discontinued because of hepatotoxicity-related ADRs in 29/50 events (58.0%), dose reduction in 4/50 (8.0%) and no change in 17/50 (34.0%).

#### Priority survey items: use of abiraterone acetate plus prednisolone in patients with hepatic impairment

Therapy with abiraterone acetate plus prednisolone was administered to 11/492 patients with hepatic impairment. In 6/11 patients (54.5%), six ADRs were reported. In patients without hepatic impairment, 164 ADRs in 125/481 patients (26.0%) were reported. There was no statistically significant difference in the occurrence of ADRs between populations with and without hepatic impairment (*P* = 0.076, Fisher’s exact test).

All ADRs in patients with hepatic impairment were abnormal hepatic function (two events), hypoesthesia, nausea, hepatotoxicity and increased alanine aminotransferase (one event for each). Of these, one patient with abnormal hepatic function was serious and recovered, and one patient with non-serious hypoesthesia did not recover. The events that led to discontinuation of abiraterone acetate were one each for abnormal hepatic function and hepatotoxicity, and that led to dose reduction was nausea. The outcomes of other events were recovering (nausea) or recovered (all other ADRs).

### Efficacy

**Figure 4.**
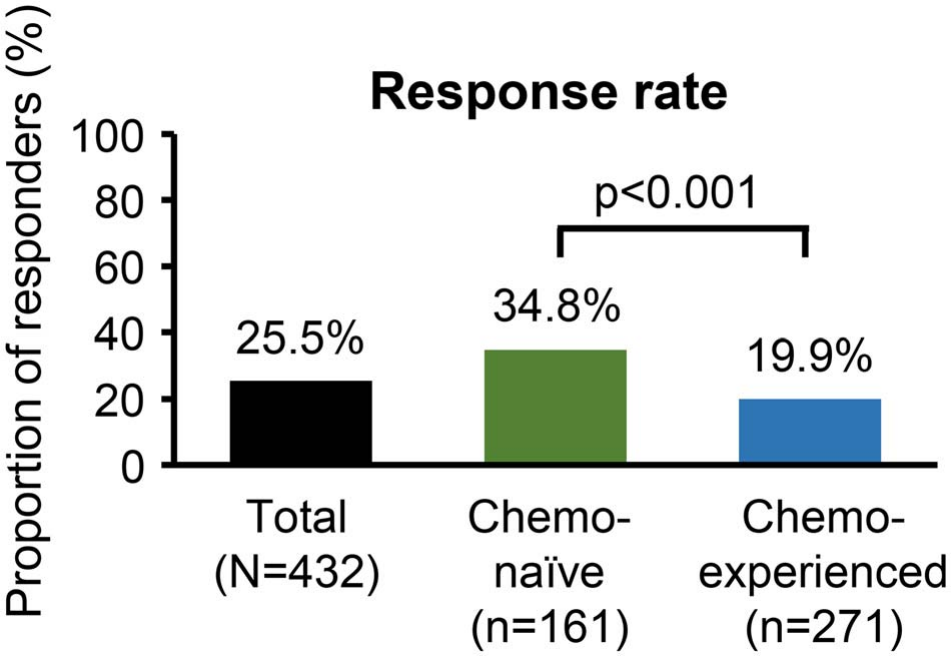
Response rate to abiraterone (*N* = 432). A responder was defined as a patient with at least a 50% reduction in PSA levels from the baseline value at the end of the observation period. Chemo-naïve, chemotherapy-naïve patients; chemo-experienced, chemotherapy-experienced patients; PSA, prostate-specific antigen.

At 12 weeks after the first administration of abiraterone acetate plus prednisone, median PSA was 16.5 (range, 0–6355) ng/ml in the overall population, 7.4 (0–3409) ng/ml in chemotherapy-naïve patients and 23.2 (0–6355) ng/ml in chemotherapy-experienced patients (efficacy analysis set; Table S3). At 12 weeks after the first administration, 110/432 had a ≥50% reduction of PSA from baseline and the overall response rate was 25.5%. The response rate was higher in chemotherapy-naïve patients (56/161 patients, 34.8%) than in chemotherapy-experienced patients (54/271 patients, 19.9%, *P* < 0.001; Fig. 4). Median duration of anti-androgen therapy before abiraterone was 1.8 (range, 0.1–12.5) years in the overall population, 1.4 (0.3–11.5) years in chemotherapy-naive patients and 2.1 (0.1–12.5) years in chemotherapy-experienced patients (efficacy analysis set). The response rate was not significantly different according to the period of anti-androgen therapy (*P* = 0.753). Patients without previous enzalutamide therapy tended to respond better than patients with enzalutamide therapy (Table S4). Patients without metastasis had a higher response rate (Table S4) than patients with metastasis.

**Figure 5.**
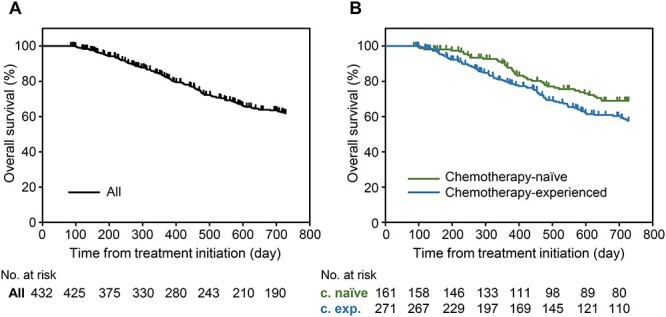
Overall survival of patients treated with abiraterone. (A) Overall survival in all patients treated with abiraterone acetate plus prednisolone (*N* = 432). (B) Overall survival in subgroups divided based on previous use of chemotherapy. c. naïve, chemotherapy-naïve patients; c. exp, chemotherapy-experienced patients.

Kaplan–Meier curves of OS are shown in Figure 5. During the study period, deaths occurred in 137/432 (31.7%) patients, including 42/161 chemotherapy-naïve patients (26.1%) and 95/271 chemotherapy-experienced patients (35.1%). The survival rates were 68.3% in the efficacy analysis set, 73.9% in chemotherapy-naïve patients and 64.9% in chemotherapy experienced patients.

## Discussion

In this study, we investigated the safety and efficacy of abiraterone acetate plus prednisolone in Japanese patients with CRPC with or without previous chemotherapy in the form of post-marketing surveillance. The data were collected from a large cohort of 492 patients over 24 months in a real-world setting. No new or unpredictable ADRs were observed.

In this study, 45.7% of patients experienced 318 AEs, of which 170 were listed as ADRs in 26.6% of patients. These data are comparable with previous studies (7,10–12). As expected, frequent ADRs were the same as those observed in pre-approval clinical studies, including hypokalemia and abnormal hepatic function (13). The most frequently occurring ADR of special interest was hepatotoxicity-related ADRs in 47 patients (50 events). Most events (49/50 events) were recovered or recovering after a dose reduction or discontinuation of abiraterone. Additionally, 11 patients with concomitant hepatic impairment were included in this study, 4 of whom had hepatotoxicity-related ADRs (four events). All events including one event of serious grade recovered. Although hepatotoxicity-related ADRs occurred frequently and required strict monitoring, they were well managed in the real-world setting.

In this study, an occurrence of hypokalemia was found in 3% of patients and one patient died. This patient was 86 years old and had concomitant diseases of angina pectoris. In this case, the patient died of hypokalemia 27 days after onset. Previously, two hypokalemia cases with grade 4 were reported in Japanese patients who had previous glucocorticoid therapy and concomitant furosemide therapy (14). Careful monitoring and the collection of more data are needed to determine patients at high risk of hypokalemia by abiraterone acetate plus prednisolone treatment.

The response rate of chemotherapy-naïve patients in this study was 34.8%. A previous Japanese phase II study (JPN-201) reported a response rate of 60.4% (11) and a global phase III study, COU-AA-302, reported a response rate of 62% (10). We cannot explain this discrepancy between previous studies and the current study. However, there is a prostate cancer therapy, which is characteristic for Japan. Before the second-generation anti-androgens were developed, first-line therapy with non-steroidal anti-androgens, such as bicalutamide and flutamide, combined with luteinizing hormone-release hormone analogues (CAB), had been recommended for patients with castration-sensitive prostate cancer (CSPC). After disease progression and castration-resistance development, the use of bicalutamide or flutamide one after another as an alternative anti-androgen therapy (AAT) became widespread in Japan (15,16). AAT has not been recommended since 2016, but it appeared that AAT was still used and flutamide was often prescribed as the subsequent agent after bicalutamide in Japan (69.5% in this study). Although our study suggested that the duration of previous anti-androgen therapy was not associated with response rate, and patients responded to abiraterone regardless of the treatment period of previous endocrine therapies in a previous post hoc analysis of the COU-AA-301 and COU-AA-302 studies (17), further evidence is warranted to determine whether previous anti-androgen therapies could be interfering with the abiraterone acetate plus prednisolone response.

Another consideration is that enzalutamide was used in nearly 20% of patients before abiraterone acetate plus prednisolone administration in this study, which indicates that it was the third most used anti-androgen following bicalutamide and flutamide, whereas enzalutamide was not preferable in the JPN-201 cohort (11). In this study as well, the response rate of patients who received pretreatment with enzalutamide was particularly low (Table S4). Several studies reported that the efficacy of abiraterone after enzalutamide treatment was poor in terms of PSA response compared with abiraterone as a first-line anti-androgen (18–20). Therefore, the lower response rate in this study might be related to the population with enzalutamide pretreatment.

Furthermore, patients with performance status 2 or higher were included in this study and excluded in the pre-approval JPN-201 or COU-AA-302 studies (10,11). It is also of note that the clinical trial was discontinued once PSA levels increased, but this is not always the case for real-world treatments. Differences in real-world settings might have caused the lower response rate in this study. Abiraterone acetate plus prednisolone may be more beneficial as first-line therapy before enzalutamide than as second-line therapy after enzalutamide. However, the response rate in chemotherapy-experienced patients was 19.9% in this study, 28.3% in the Japanese phase II study JPN-202 (12) and 29.1% in the global phase III study COU-AA-301 (7), which were comparable.

This study had several limitations. We conducted an observational, post-marketing surveillance study without any control groups; therefore, comparisons could not be made. Because of data availability, we had to perform categorical analysis for the PSA response rate, which is less informative than, for example, Kaplan–Meier analysis to estimate time to PSA progression. Furthermore, patient demographics were more diverse than in controlled clinical studies, which might have interfered with interpreting the safety and efficacy data; however, this reflects real-world clinical situations. Of note, the use of abiraterone may have been changed after closing the data collection of this study. For example, the use of abiraterone for patients with high-risk, metastatic, CSPC has been approved (21). Therefore, this study may not reflect the most current clinical practice.

In conclusion, the present study confirmed the safety and efficacy profiles of abiraterone acetate plus prednisolone reported in pre-approval clinical studies in a real-world clinical setting. Further monitoring is important to update the profiles with the most current use of abiraterone acetate plus prednisolone and to manage treatment-associated AEs.

## Supplementary Material

Koroki_et_al_Supplementary_tables_hyab077
