# Supplementary material for: Safety and efficacy of abiraterone acetate plus prednisolone in patients with castration-resistant prostate cancer: a prospective, observational, post-marketing surveillance study
Source: Jpn J Clin Oncol. 2021 May 29;51(9):1452–61. doi: 10.1093/jjco/hyab077 (PMC8405844; doi:10.1093/jjco/hyab077)
Supplement: Koroki_et_al_Supplementary_tables_hyab077 [file koroki_et_al_supplementary_tables_hyab077.doc]

# Supplementary Tables

## Table S1. Administration status of abiraterone at week 52 (Safety analysis set, N=492)

| Abiraterone administration | Number of patients, n (%) |
| --- | --- |
| Continued | 115 (23.4) |
| Discontinued | 377 (76.6) |
| <12 weeks | 152 (40.3)* |
| ≥12 weeks, <24 weeks | 108 (28.6)* |
| ≥24 weeks, <36 weeks | 60 (15.9)* |
| ≥36 weeks, <52 weeks | 46 (12.2)* |
| ≥52 weeks | 11 (2.9)* |

* Proportion in discontinued patients

## Table S2. Summary of adverse events leading to treatment modification in 2 or more patients (Safety analysis set, N=492)

|  | Number of patients, n (%)  Number of events, n (%) |
| --- | --- |
| Any AE | 225 (45.7) |
| Any serious AE | 160 (32.5) |
| AE leading to treatment discontinuation | 88 (17.9)* |
| Hepatic function abnormal | 19 (3.9) |
| Prostate cancer | 17 (3.5) |
| Decreased appetite | 5 (1.0) |
| Hepatotoxicity | 4 (0.8) |
| Hypokalemia | 4 (0.8) |
| Malaise | 3 (0.6) |
| Pneumonia | 3 (0.6) |
| Anaemia | 2 (0.4) |
| Drug eruption | 2 (0.4) |
| Fatigue | 2 (0.4) |
| Hyperglycemia | 2 (0.4) |
| AE leading to treatment dose reduction | 10 (2.0) * |
| Hepatic function abnormal | 4 (0.8) |
| Nausea | 2 (0.4) |
| Dizziness | 2 (0.4) |

*Including patients who experienced 2 or more events

## Table S3. Prostate-specific antigen values at baseline and at week 12 (Efficacy analysis set, N=432)

| Prostate-specific antigen [ng/mL] | Total, N=432 | Chemotherapy-naïve, N=161 | Chemotherapy-experienced, N=271 |
| --- | --- | --- | --- |
| Baseline |  |  |  |
| Median (range) | 17.4 (0.01 – 2667) | 13.0 (0.03 – 1819) | 20.3 (0.01 – 2667) |
| Unknown, n | 0 | 0 | 0 |
| At week 12 |  |  |  |
| Median (range) | 16.5 (0 – 6355) | 7.4 (0 – 3409) | 23.2 (0 – 6355) |
| Unknown, n | 56 | 22 | 34 |

## Table S4. Response rate to abiraterone (Efficacy analysis set, N=432)

| Responder* | Total, N=432,  n (%) | | | Chemotherapy-naïve, N=161,  n (%) | | Chemotherapy-experienced, N=271,  n (%) | |  |
| --- | --- | --- | --- | --- | --- | --- | --- | --- |
| Responder, total | 110 (25.5) | | | 56 (34.8) | | 54 (19.9) | |  |
| Previous enzalutamide therapy | | | | | | | |  |
|  | Yes  n=78 | | No  n=354 | Yes  n=26 | No  n=135 | Yes  n=52 | No  n=219 |  |
| Responder | 5 (6.4) | 105 (29.7) | | 1 (3.8) | 55 (40.7) | 4 (7.7) | 50 (22.8) |  |
| Metastasis |  | | |  | |  | |  |
|  | Yes  n=347 | | No  n=85 | Yes  n=128 | No  n=33 | Yes  n=219 | No  n=52 | |
| Responder | 80 (23.1) | | 30 (35.3) | 40 (31.3) | 16 (48.5) | 40 (18.3) | 14 (26.9) | |

* A responder was defined as a patient with at least a 50% reduction in prostate-specific antigen levels from the baseline value at week 12.
